# Spatial proteomics identifies altered thick ascending limb cells as drivers of kidney disease through uromodulin retention

**DOI:** 10.64898/2026.10.09.756985

**Published:** 2026-10-12

**Authors:** Azuma Nanamatsu, George J. Rhodes, William S. Bowen, Angela R. Sabo, Shehnaz Khan, Mahla Asghari, Dillen L. Wischmeier, Daria Barwinska, Takashi Kudo, Azusa Tomioka, Hiroaki Sakaue, Atsushi Kuno, Ana C. Ricardo, James P. Lash, Katherine R. Tuttle, Pierre C. Dagher, Jonathan Himmelfarb, Kaice A. LaFavers, Luca Rampoldi, Tarek M. El-Achkar

## Abstract

Chronic kidney disease (CKD) is increasingly prevalent and is a leading cause of mortality. Recent transcriptomic studies have identified the persistence of injury-induced renal tubular epithelial cell states, yet the cell populations that drive CKD remain unresolved. Uromodulin (UMOD) is the most abundant urinary secreted protein expressed exclusively in thick ascending limb (TAL) epithelial cells in the kidney. UMOD is strongly associated with CKD risk in genome-wide association studies, suggesting a broader, yet unexplored role of UMOD and TAL cells in CKD pathogenesis. Here, using spatial proteomics, we identified an injury-induced TAL cell state marked by intracellular UMOD accumulation. Spatially resolved multimodal integration revealed that UMOD accumulation occurred without transcriptional upregulation. Impaired urinary UMOD secretion further supported pathological intracellular retention. These altered TAL cells with UMOD retention exhibited inflammatory signaling, formed profibrotic niches enriched with M2-like macrophages across renal compartments, and were associated with CKD progression in multiple experimental models. Reducing the burden of UMOD retention diminished altered TAL cells and their profibrotic microenvironments, and attenuated CKD development. A similar altered TAL state was identified in human kidney disease and was associated with worse kidney function. Together, we identify pathological UMOD retention and altered TAL cells as key drivers of CKD development and potential therapeutic targets. This study also highlights that protein-level alterations can redefine disease-associated cell states beyond transcript profiling.

